# Sources of nutrition information and level of nutrition knowledge among young adults in the Accra metropolis

**DOI:** 10.1186/s12889-018-6159-1

**Published:** 2018-11-29

**Authors:** Esi Yaabah Quaidoo, Agartha Ohemeng, Margaret Amankwah-Poku

**Affiliations:** 1 Department of Nutrition and Food Science, College of Basic and Applied Sciences, University of Ghana, Legon, Accra, Ghana; 2 Department of Psychology, College of Humanities, University of Ghana, Legon, Accra, Ghana

**Keywords:** Sources of nutrition information, young adults, nutrition knowledge, nutritional behaviours

## Abstract

**Background:**

Acquiring accurate and adequate nutrition information is important as it could inform nutritional choices positively and promote the maintenance of a healthy nutritional status. This study assessed a sample of young adults’ nutrition knowledge and identified where they gather information from to guide nutritional choices.

**Method:**

This was a cross-sectional study involving young adults (*N*=192) between 18 to 25 years recruited at shopping areas in the Accra Metropolis of Ghana. A pretested questionnaire was used to collect information on demographic characteristics, sources of nutrition information and basic nutrition knowledge. Pearson chi-square test was used to identify possible differences between high and low scorers of the knowledge assessment in terms of their nutrition information acquisition behaviours and logistic regression was conducted to ascertain whether source of nutrition information was related to participants’ nutrition knowledge.

**Results:**

Online resources were the most popular source (92.7%) used to seek information on nutrition among study participants, and healthcare professionals were perceived to be the most reliable source of nutrition information. Additionally, participants who used healthcare professionals as a source of nutrition information were 61% (95% CI: 0.15-0.99) more likely to have a high nutrition knowledge than participants who did not consult healthcare professionals for nutrition information.

**Conclusion:**

Online resources serve as a very common source of nutrition information for young adults. Thus, healthcare professionals may need to adopt this as a useful channel to circulate trustworthy nutrition information to this age group.

**Electronic supplementary material:**

The online version of this article (10.1186/s12889-018-6159-1) contains supplementary material, which is available to authorized users.

## Background

With the relative independence that accompanies young adulthood, individuals who previously had little to no control over their nutritional choices and how they conduct their nutrition-related behaviours shift to having prime control over themselves since many are not under parental or caretaker scrutiny [14, 19, 20]. As such, many young adults adopt unhealthy nutritional habits such as increased consumption of alcoholic beverages [19], frequent patronage of fast food and convenience food [3, 10] and low personal meal preparation [9, 14]. Awareness of the importance of practicing healthy nutritional behaviours such as reading labels before purchasing packaged food products could be propagated widely through the use of young adults’ frequented sources of nutrition information.

Adequate nutrition knowledge has been described as having an awareness of practices and concepts related to nutrition including adequate food intake and wellbeing, food intake and disease, foods signifying key sources of nutrients and dietary guidelines and references [12]. Some studies have suggested that adequate level of nutrition knowledge is related to optimal nutritional behaviours [2, 8, 12]. Thus, access to credible nutrition information may serve as the basis for appropriate practices. For instance, an individual with adequate knowledge on nutrition stands a better chance of differentiating nutrition facts from nutrition fads [1]. The sources of nutrition information used in various communities, among different demographics is important to know since highly patronized sources of information in a society can be used as an effective tool to disseminate accurate nutrition information to the masses. Information disseminated through old (traditional media) and new (online resources) media play a role in determining nutrition choices as they market ideas and products that have the ability to influence behaviours [16]. Yet studies that have reported nutrition information acquisition behaviours of young adults from developing countries are limited. Data on nutrition information acquisition behaviours is needed to develop community-specific interventions that can promote a lifespan of good nutritional habits and hence good health. This data is especially needed at this critical life stage because of the new experiences many young adults face such as putting together their own meals; nutrition habits acquired at this stage will greatly contribute to their quality of life in later years.

Common sources of nutrition information identified in the literature include the internet, family members and friends, television, and books [4, 6, 13, 15, 22]. Although studies have indicated that online resources are popular, there are differences with respect to its usage and perceived reliability among different samples. Thus, more information is needed on health information acquisition behaviours, particularly in economically emerging countries where internet use is fast becoming a common tool for information acquisition.

This study therefore sought to describe the sources of nutrition information used by a sample of young adults residing in an urban setting in Ghana.

## Methods

The study took place in the Accra Metropolis in the Greater Accra Region of Ghana. Accra has many shopping areas which are heavily populated with young individuals; hence the researcher deemed it an appropriate venue to sample a diverse group of young adults. Two shopping centres (Accra Mall and Makola Market) were randomly selected from a list of shopping areas (malls and markets) that were located in Accra at the time of study. The target population was young adults between the ages of 18 to 25 who had completed at least their junior high school education, and were residing in the Accra metropolis at the commencement of the study.

Participants were recruited using convenience sampling method at both study sites. The researcher and her field assistants briefly engaged young adults to ascertain if they met all the study’s inclusion criteria. The objectives of the study were thoroughly explained to prospective participants and they were enrolled into the study only after they had signed an informed consent form.

The questionnaire that was developed for this study (see Additional file 1) was interviewer administered and approximately 20 minutes was spent on each participant. A total of 298 young adults were approached at these shopping areas and 192 completed the questionnaire. This represents a response rate of 64.4%. The questionnaire was pretested at another shopping mall and market place in Accra among 16 young adults. Questions in the pretest included demographic information, an open-end question on all of a participant’s sources of nutrition information as well as an assessment of basic knowledge on nutrition. Based on the findings from the pretest some modifications were made to the questionnaire: participants’ responses (when categorized according to themes) were family members, online resources, friends and peers, healthcare professionals and traditional media. Therefore, the question posed on where sources of nutrition information were sought was made close-end, citing five sources of information in a table i.e. family members, online resources, friends and peers, healthcare professionals and traditional media, making the questionnaire easier to complete. A Likert scale with options: ‘always’, ‘rarely’ and ‘never’, was provided for participants to identify how often they turn to these sources when they seek information regarding their nutrition. Perceived reliability of these sources of information was also assessed using a Likert scale with options ‘unreliable’, ‘fairly reliable’ and ‘very reliable’. The questionnaire was also used to obtain demographic information as well as an assessment of basic nutrition knowledge. The basic nutrition knowledge section consisted of fifteen close ended questions. The questions posed to participants covered knowledge on the nutrition facts panels, knowledge on appropriate daily dietary and lifestyle habits. The lowest score a participant could obtain was 0 and the highest was 15 points.

Ethical approval for this study was obtained from the Ethics Committee for the College of Basic and Applied Science (ECBAS), University of Ghana (ECBAS 006/16-17). Permission to collect data from the Accra mall was obtained from management of the mall. Makola market is an open market, thus this process was not required.

Statistical package for social scientists (SPSS) 16.0 software was used to analyze all data at 95% confidence interval. Demographic data and frequency of use of sources of nutrition information was taking through descriptive analysis. The mean score for nutrition knowledge of participants was calculated and this was used in creating a categorical variable for nutrition knowledge: scores below the mean were considered low whilst scores above or equal to the mean were considered high. A Pearson chi-square test was conducted to identify possible differences between high and low basic nutrition scorers and their information acquisition behaviours. Logistic regression models were used to ascertain whether any of the sources was/were related to the level of nutrition knowledge.

## Results

**Fig. 1:**
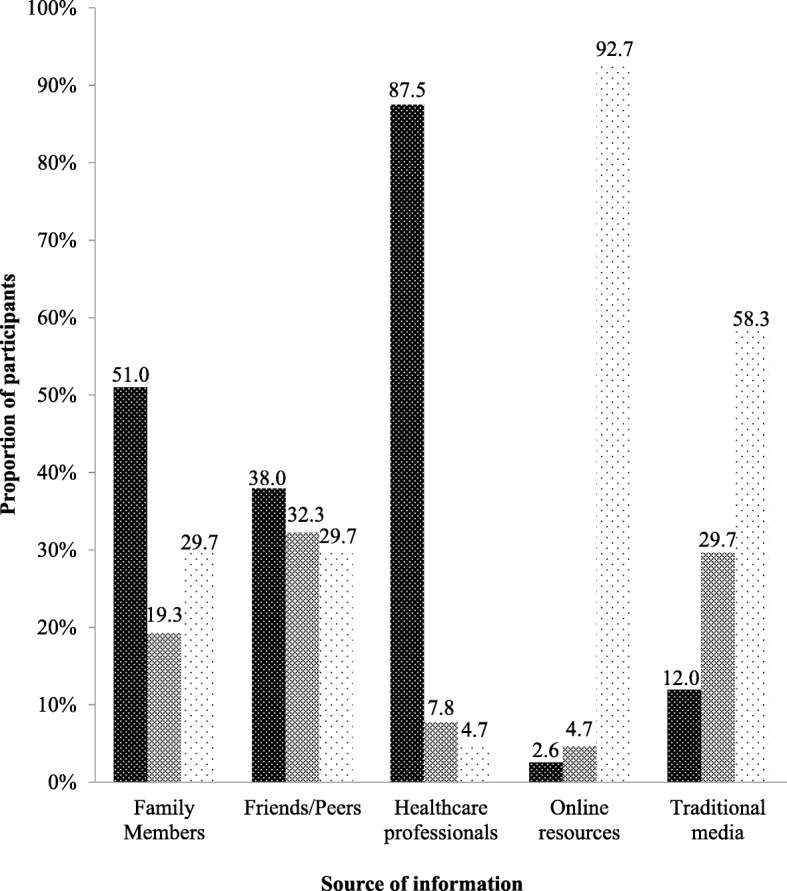
Sources of participants’ nutrition information (*N*=192). Dense polka dot pattern indicates participants who never used that information source. Brick pattern indicates participants who rarely used the information source. Thin polka dot pattern indicates participants who always used that source.

**Fig. 2:**
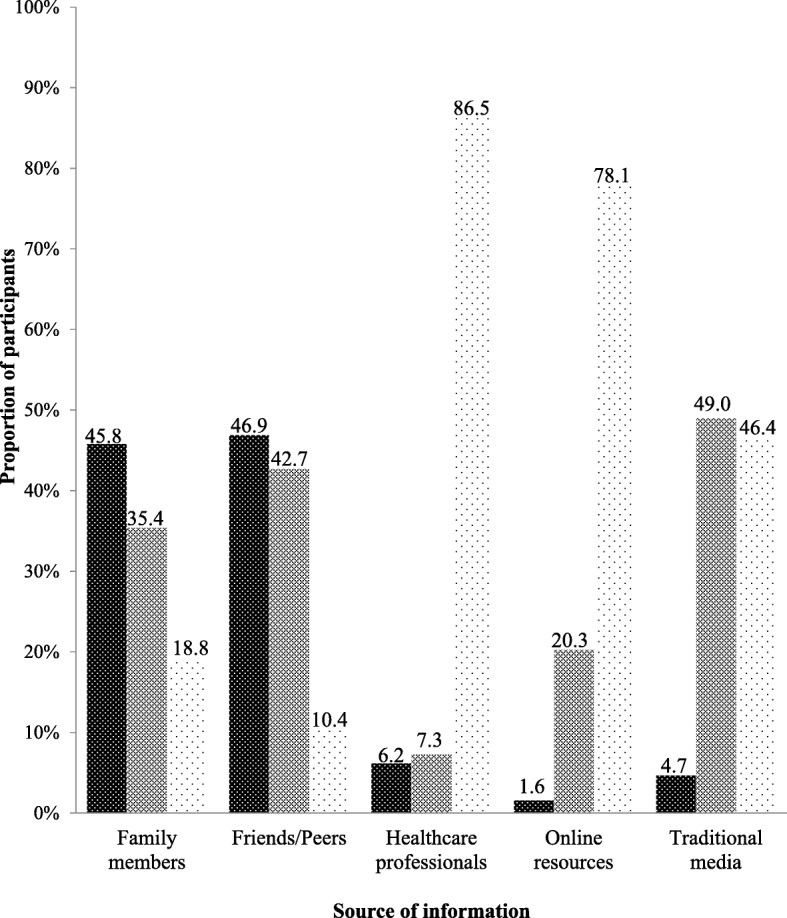
Perceived reliability of sources of nutrition information (*N*=192). Brick pattern indicates the proportion of study participants who considered information source as fairly reliable. The thin polka dot pattern indicates the proportion of study participants who considered the source to be very reliable, whilst the thick polka dot pattern indicates the proportion of participants who perceived the source to be unreliable.

One hundred and ninety-two participants took part in this study, about half (51%) of which were females (Table 1). The mean age was 21.8 (2.2) years and most of the participants were students (66.1%) and had completed a senior high school level education. Figure 1 illustrates the various sources of nutrition information and the extent to which these sources were used by study participants when seeking information on nutrition. Online resources were the most popular source used to seek information on nutrition among study participants (92.7%). Traditional media was the second most used source with 58.3% of participants always seeking nutrition information from television programmes, radio programmes and newspaper articles. Healthcare professionals, including nutritionists and dietitians were the least used source of nutrition information. Majority of the participants (87.5%) had never sought nutrition information from healthcare professionals although majority (86.5%) perceived nutrition information from healthcare professionals to be very reliable (Fig. 2). Additionally, online resources were perceived by most participants (78.1%) as very reliable source of nutrition information. Interestingly, nutrition information from friends and peers was perceived by almost half of participants (46.9%) as the most unreliable source of nutrition information.

**Table 1:** Demographic characteristics of participants (*n* = 192)

	Mean (SD)	*n* (%)
Age (years)	21.8 (2.2)	
Sex		
Male		94 (49.0)
Female		98 (51.0)
Ethnicity		
Akan		94 (49.0)
Ewe		45 (23.4)
Ga-Adangbe		40 (20.8)
Northerner		13 (6.8)
Occupation^a^		
Student		127 (66.1)
Services/Sales workers		23 (12.0)
Professionals		19 (9.9)
Other^b^		18 (9.4)
Unemployed		5 (2.6)
Highest educational qualification		
Senior high school		127 (66.1)
Post-secondary school^c^		44 (23.0)
Junior high school		21 (10.9)

^a^Aside from ‘student’, occupation of participants was categorized based on the International Standard Classification of Occupations (ISCO) [7]

^b^Other occupations included Crafts and related trades workers, Clerical support workers, and Elementary Occupation

^c^Includes clerical, vocational, polytechnic and university institutions

**Fig. 3:**
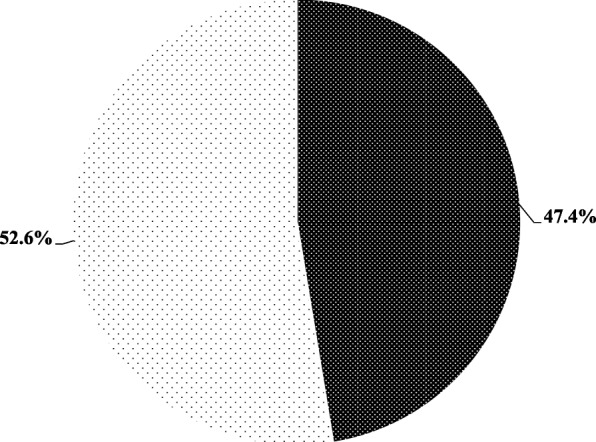
Basic nutrition knowledge scores of study participants; (*N*=192). Shows the categorization of study participants based on the nutrition knowledge assessment. The thin polka dot pattern indicates the proportion of study participants who scored high on the basic nutrition knowledge assessment, whilst the thick polka dot pattern indicates the proportion of participants who scored low on the basic nutrition knowledge assessment.

Figure 3 shows the basic nutrition knowledge scores of participants. The mean basic nutrition knowledge score for the entire sample was 9.7(1.8) out of 15.0 points. Although about half of the participants were categorized as high scorers on the basic nutrition knowledge assessment, more than half (62.5%) of the participants in this study did not know what the nutrition facts panel was. Of those who indicated some knowledge of nutritional facts panel, less than half (44.3%) of participants indicated that they had some understanding of the nutrition information provided on their packaged foods.

Comparing the means, participants who consulted health professionals for nutrition information (10.5 vs 9.6, *p* = 0.02) and those who used online resources (9.7 vs 8.8, *p* = 0.04) scored higher marks on the nutrition knowledge assessment, compared to those who did not use these resources. There were no differences in the scores when the other identified sources of information were considered. Online resource was not included in the logistic regression analyses due to the fact that very few participants (*n* = 5) did not use it and all of these scored low on the knowledge assessment (Table 2), therefore there was very little variation. After adjusting for age, education level, and sex, those who consulted healthcare professionals were less likely (adjusted OR = 0.39; 95% CI: 0.15, 0.99) to obtain low scores on the nutrition knowledge assessment, compared to those who did not consult professionals (Table 3). None of the other sources of nutrition information identified was related to the level of nutrition knowledge.

**Table 2:** Bivariate analysis of sources of nutrition information and nutrition knowledge (*N* = 192)

Source of nutrition information	Basic Nutrition Knowledge^a^		
	Low Score	High Score	*p*-value
Family members			
Yes	44 (48.4)	49 (48.5)	0.982
No	47 (51.6)	52 (51.5)	
Friends/Peers			
Yes	56 (61.5)	63 (62.4)	0..905
No	35 (38.5)	38 (37.6)	
Healthcare professionals			
Yes	7 (7.7)	17 (16.8)	0.056
No	84 (92.3)	84 (83.2)	
Online resources			
Yes	86 (94.5)	101 (100)	0.017^#^
No	5 (5.5)	0 (0)	
Traditional media			
Yes	80 (97.9)	90 (89.1)	0.795
No	11 (12.1)	11 (10.9)	

^a^The mean score for nutrition knowledge of participants was calculated and this was used in creating a categorical variable for nutrition knowledge: scores below the mean were considered low whilst scores equal to or above the mean were considered high

^#^*p*-value < 0.05

**Table 3:** Association between sources of nutrition information and nutrition knowledge, based on logistic regression models^b^

Source of information^a^	Low Nutrition Knowledge^c^	
	OR	95% confidence interval
Family	1.078	0.601–1.933
Friends	1.000	0.545–1.836
Healthcare professionals	0.385^*^	0.150–0.990
Traditional media^d^	0.854	0.347–2.100

^a^Using binary logistic regression, the relationship between use of online resources and nutrition knowledge could not be determined because of the low level of variation in its use by participants

^b^The factors accounted for in the models were age, sex, and educational level

^c^Low score means below the mean of the sample. The maximum that one could obtain is 15 points

^d^This refers to television and radio programmes, as well as newspapers

*p-value < 0.05

## Discussion

Even though a little over half of the study’s participants had high scores on the basic nutrition knowledge assessment, over half of participants indicated that they did not understand nutrition concepts such as how to interpret the information provided on nutritional facts panels. The usage of nutrition facts panels is associated with making healthy food choices [18]. Nutrition facts panels are the key means of offering information on the nutritional content of packaged food products consumers purchase and as such it is vital that information provided is actually referred to and understood [11]. Hayford, Sakyi-Dawson & Steiner-Asiedu, [5], conducted a study on the knowledge and use of food labels among Ghanaians and found that although participants felt that nutrition information provided on food packages was important, majority did not know how to interpret the information on nutrition labels. Facts panel usage was also low among participants in the present study, affirming the need for nutrition facts panel usage and interpretation education.

There is a myriad of nutrition information available from different sources and varying in credibility but where a young person looks for nutrition information depends partly on the social context. Online resources were the most patronized source by participants in the present study, whereas an Iranian study found television programmes to be the first go-to for health information, followed by family members and/or close friends, books and public libraries [4]. Online resources may have been the most patronized source of nutrition information due to the increased improvements of telecommunication technologies in Ghana. More Ghanaians have access to the internet than previous years when telecommunication infrastructure was not as advanced [13]. Many young people have the internet at their service on several portable digital devices, anywhere and at any time and this has enabled more opportunities to look up information rapidly and conveniently. Majority of the participants in the present study considered online resources as very reliable which is consistent with findings by Obasola & Agunbiade, [13]. This however contradicts Zhang’s, [22], report on young adult Americans not perceiving online resources as wholly reliable even though they used online resources regularly when looking for health-related matters. There appears to be a difference between the way young adults from developing countries and developed countries identify quality health information. According to Zhang [22], his study participants felt it was not advisable to take health information posted on online platforms such as social networking sites as accurate.

Traditional media (television, radio, newspapers, etc) was the second most used source for acquiring nutrition information in the present study. Many participants in this study indicated that nutrition information they acquired from traditional media came about passively. The information disseminated by traditional media is often made for heterogeneous audiences and not tailored to meet specific needs of individuals unlike online resources which host communities of like-minded individuals sharing health information of interest to specific people [16]. Thus, information from traditional media can be impersonal and this may be the reason why many participants in this study did not perceive nutrition information from this source as very reliable. This study also identified friends and peers as an important source of nutrition information, similar to findings from a study on health seeking behaviours by Percheski & Hargittai, [15]. Some participants indicated that after consulting friends they would use the internet to verify whatever information their friends or peers gave them. Thus, online resources were considered to be of higher quality than ideas from friends for these participants. On the other hand, Percheski & Hargittai’s study reported that the internet served purely as a complementary source of information and not as a substitute for other sources such as healthcare professionals, family members and peers among their sample.

Healthcare professionals were the least used source of nutrition information although they were perceived as the most reliable source, even more so than online resources. Obasola & Agunbiade, [13], pointed out in their study that in many developing countries there are firmly held cultures of self-care practices. When it comes to health, only severe malaise would push one to seek professional medical assistance. A random sample of medical facilities in Accra indicated that to see a registered dietitian or nutritionist in a medical facility would attract a fee ranging from ten to thirty Ghana cedis i.e. 2.28 dollars to 6.83 dollars [17]. Many young adults have limited financial freedom and may not be willing to part with that amount of money for one session with a healthcare professional while they can purchase data at a cheaper price and go online for nutrition information. Healthcare professionals are trained to be literate in health matters and have information about the body and the cause of disease [21]. This includes an understanding of nutritional behaviours and the health implications of such behaviours. Study participants who did use healthcare professionals as a source of nutrition information were less likely to score low on the nutrition knowledge assessment, indicating that interaction with healthcare professionals gave those participants the advantage of having an awareness of practices and concepts related to nutrition as compared to the participants who did not turn to healthcare professionals for nutrition information. It would be worthwhile for healthcare professionals to package health information in an easily accessible manner using online resources to reach young adults and also sensitize young individuals.

## Study limitations

These findings should be interpreted with some level of caution. The study establishes possible associations, not causality. Although the researchers employed simple random sampling in selecting the study centers (Accra mall and Makola Market), convenience sampling used in selecting the participants at these points in order to meet sample size requirement within the stipulated data collection period. This limits the level to which findings can be generalized. Again, not all factors that could possibly influence nutrition information seeking behavior, such as health status, were not assessed and accounted for in the current study. Additional information on timing and actual quality of the nutrition information received from any of the sources was not assessed.

## Conclusion

In this study among young adults in the Accra Metropolis, although healthcare professionals were perceived to be the most reliable source of nutrition information and was associated with higher nutrition knowledge, only few participants consulted them. On the other hand online resources serve as a very important source of nutrition information for this age group. Further studies should be carried out to identify the quality of online information used by individuals within this age group, in order to ensure nutrition information offered is credible. Nutrition education, such as nutrition facts panel reading and interpretation, should be introduced preferably to adolescents before they enter young adulthood where they experience more freedom to make their own choices than prior life stages.

## Additional file

Additional file 1: Research Questionnaire. Document is a presentation of questions that study participants responded during the study and the basis of the data presented in this manuscript. (DOCX 16 kb)

## Abbreviations

ECBAS: Ethics Committee for the College of Basic and Applied Science
SPSS: Statistical package for social scientists
