# Supplementary material for: Sources of nutrition information and level of nutrition knowledge among young adults in the Accra metropolis
Source: BMC Public Health. 2018 Nov 29;18:1323. doi: 10.1186/s12889-018-6159-1 (PMC6267800; doi:10.1186/s12889-018-6159-1)
Supplement: Supplementary file 1 — Research Questionnaire. Document is a presentation of questions that study participants responded during the study and the basis of the data presented in this manuscript. (DOCX 16 kb) [file 12889_2018_6159_MOESM1_ESM.docx]

## Research Questionnaire

Code of Respondent: Date of interview:

Thank you for taking the time to fill this short questionnaire. We will keep your responses confidential so please answer these questions as honestly as possible. There is no right or wrong answer.

1. What is your gender?
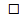
Male
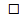
Female
2. What is your ethnicity …………………………………..
3. What is your occupation? …………………………………………
4. How old are you? …………………… Date of birth: ………………………………
5. What is your marital status ……………………………….
6. What is your highest education qualification ……………………………………………

**Acquisition of Nutrition Information:**

1. Where do you get information on nutrition from? For example, if you want information on the benefits of eating yogurt? **Please tick**

|  | 0=Never | 1= Rarely | 2= Sometimes | 3=Always |
| --- | --- | --- | --- | --- |
| Family members |  |  |  |  |
| Friends & peers |  |  |  |  |
| Healthcare professionals (e.g. nutritionists, dietitians, doctors etc.) |  |  |  |  |
| Online resources (e.g. Google searches, YouTube etc.) |  |  |  |  |
| Traditional media (e.g. Radio, television, newspapers etc.) |  |  |  |  |

1. How reliable do you think the information from these sources is? **Please tick**

|  | 0= Unreliable | 1= Fairly reliable | 2= Very reliable | 3= Accurate |
| --- | --- | --- | --- | --- |
| Family members |  |  |  |  |
| Friends & peers |  |  |  |  |
| Healthcare professionals (e.g. nutritionists, dietitians, doctors etc.) |  |  |  |  |
| Online resources (e.g. Google searches, YouTube etc.) |  |  |  |  |
| Traditional media (e.g. Radio, television, newspapers etc.) |  |  |  |  |

**Please write the number (code) attached to your option in the brackets**:

1. Do you know what the facts panel on food packages is?

0= No 1= Yes **[ ]**

1. How often do you read the information provided on food packages?

0= Never 1= Less Frequently 2= Frequently 3= Always **[ ]**

1. Do you understand what you read on food packages?

0= Never 1= Less Frequently 2= Frequently 3= Always **[ ]**

1. How many full meals do you think a person must eat in a day?

0= More than 3 1= 3 a day 2=2 a day 3= 1 a day 4= I do not know **[ ]**

1. How many sachets of water do you think a person should drink in a day?

0= 6 and above 1= 5-4 sachets 2= Less than 3 sachets 3= I do not know **[ ]**

**Please use the numbers below to indicate how much you agree or disagree with the following statements.**

**0=** neutral **1=** strongly disagree **2=** disagree  **3=** agree **4=** strongly agree

1. Eating breakfast every day is important **[ ]**
2. Eating fruits every day is important **[ ]**
3. Eating fish or chicken or meat every day is important **[ ]**
4. Drinking alcohol is bad for me **[ ]**
5. Smoking is bad for me **[ ]**
6. Eating vegetables every day is good for me **[ ]**
7. Exercise is good for me **[ ]**
8. Eating a lot of starchy foods, like yam, cassava and rice will make me fat **[ ]**
9. Eating different foods every day gives me all the vitamins and minerals I need **[ ]**
10. It is okay for me to skip meals **[ ]**
